# Assessment of disaster preparedness among emergency departments in Italian hospitals: a cautious warning for disaster risk reduction and management capacity

**DOI:** 10.1186/s13049-016-0292-6

**Published:** 2016-08-15

**Authors:** Matteo Paganini, Francesco Borrelli, Jonathan Cattani, Luca Ragazzoni, Ahmadreza Djalali, Luca Carenzo, Francesco Della Corte, Frederick M. Jr. Burkle, Pier Luigi Ingrassia

**Affiliations:** 1 CRIMEDIM – Research Center in Emergency and Disaster Medicine, Università del Piemonte Orientale, Via Lanino 1, 28100 Novara, Piemonte Italy; 2 Emergency Medicine Residency Program – Department of Medicine, Azienda Ospedaliera Università di Padova, Padova, Veneto Italy; 3 Emergency Medicine Residency Program – Department of Medicine, Università di Modena e Reggio Emilia, Modena, Emilia Romagna Italy; 4 School of Medicine, Università del Piemonte Orientale, Novara, Piemonte Italy; 5 Harvard Humanitarian Initiative, Harvard University, Cambridge, MA USA

**Keywords:** Hospital disaster preparedness, Hospital plan, Emergency department, Disaster

## Abstract

**Study hypothesis:**

Since the 1990s, Italian hospitals are required to comply with emergency disaster plans known as Emergency Plan for Massive Influx of Casualties. While various studies reveal that hospitals overall suffer from an insufficient preparedness level, the aim of this study was to better determine the preparedness level of Emergency Departments of Italian hospitals by assessing the knowledge-base of emergency physicians regarding basic disaster planning and procedures.

**Methods:**

A prospective observational study utilized a convenience sample of Italian Emergency Departments identified from the Italian Ministry of Health website. Anonymous telephone interviews were conducted of medical consultants in charge at the time in the respective Emergency Departments, and were structured in 3 parts: (1) general data and demographics, (2) the current disaster plan and (3) protocols and actions of the disaster plan.

**Results:**

Eighty-five Emergency Departments met inclusion criteria, and 69 (81 %) agreed to undergo the interview. Only 45 % of participants declared to know what an Emergency Plan for Massive Influx of Casualties is, 41 % believed to know who has the authority to activate the plan, 38 % knew who is in charge of intra-hospital operations. In Part 3 physicians revealed a worrisome inconsistency in critical content knowledge of their answers.

**Conclusions:**

Results demonstrate a poor knowledge-base of basic hospital disaster planning concepts by Italian Emergency Department physicians-on-duty. These findings should alert authorities to enhance staff disaster preparedness education, training and follow-up to ensure that these plans are known to all who have responsibility for disaster risk reduction and management capacity.

**Electronic supplementary material:**

The online version of this article (doi:10.1186/s13049-016-0292-6) contains supplementary material, which is available to authorized users.

## Background

Disasters are events capable of bringing a heavy burden in terms of morbidity and mortality. On the basis of published reports, since 1990, 1.6 million people worldwide have died because of disasters, making for an approximate average of 65,000 deaths per year [1]. Italy is a country prone to catastrophic events, especially earthquakes and floods. In the last 60 years, 25 major events have occurred including earthquakes, landslides and floods accounting for the majority of deaths, injured and homeless [2–4].

During disasters that result in a patient surge, hospitals are expected to function as a safe environment for personnel and provide essential medical care to the casualties [5]. However, various studies show that hospitals overall suffer from an insufficient level of preparedness [6, 7]. This is consistent with results of previous studies which revealed weakness in hospital disaster management, including confusion over roles and responsibilities, poor communication, lack of planning, and suboptimal training [6, 8, 9]. These findings, supported by an increased global awareness of disaster risk reduction and management, have led to the development of international strategies to improve disaster preparedness and resiliency. The Sendai Framework for Disaster Risk Reduction 2015 – 2030, recently adopted by 187 countries including Italy, stress, amongst the other priorities for action, the need to strengthen functional disaster risk prevention and reduction measures in critical facilities, including hospital emergency departments [10].

Preparedness is defined as actions taken to effectively anticipate, respond to, and recover from the impact of likely or current hazard events or conditions [11]. A hospital disaster plan (HDP) is defined as the systematic procedures that clearly detail what needs are to be performed, how, when, and by whom before and after the time an anticipated disaster event occurs [12]. In Italy, since the 1990s, hospitals are required to adopt an emergency disaster plan for external disasters, known as Emergency Plan for Massive Influx of Casualties (PEMAF acronym in Italian), in order to face a sudden patient surge [13–15].

It is crucial to hospital disaster preparedness that the PEMAF be known and understood by those professionals who will apply it. Emergency Department employees are usually the first hospital responders in the event of disasters and are the front lines of preparedness. The aim of our study was to better determine the current preparedness of Emergency Departments of Italian hospitals by assessing the level of knowledge of front line emergency physicians-on-duty regarding basic disaster planning and procedures under the governmental PEMAF.

## Methods

A cross-sectional study was used in this research and conducted from September 1, 2015, to September 30, 2015. The study group consisted of the medical consultants working at Emergency Departments (EDs) of hospitals throughout Italy. The selection of participating hospitals represents a convenience sample identified from listings available at the Italian Ministry of Health website [16]: specialized hospitals (e.g. in orthopedics, pediatrics or obstetrics and gynecology), hospitals without a complete contact list or clear informations about their level were excluded. The classification of EDs was based on the required level of care according to the criteria elicited in the Italian law D.Lgs. 70/2015 that considers, among others, factors as structures’ bed number, population density and needs of critical care / specialized surgery facilities in that geographical area [17]; according to this, a first level ED has at least internal medicine, general surgery, critical care and cardiology intensive care units, instead a second level ED has also specialized surgery (e.g. neurosurgery, cardiac surgery) and specialized critical care units in addition. Anonymous pre-structured telephone interviews were conducted during day shift with medical consultants in charge in the EDs who agreed to participate in the study. The following exclusion criteria were considered: (a) private hospitals, or (b) more than three tentative calls without answer, or (c) interruption due to sudden urgencies, or (d) clear refusal to participate the study.

### Interview tool

A standardized structured interview was developed and consisted of the following content sessions:

(1) General data and demographics of the interviewed consultant;

(2) general knowledge about the PEMAF disaster plan, including three dichotomous questions (YES or NO), followed by a simulated call from Emergency Medical Service operation center which provided a brief description of a disaster incident. The participants responded to the METHANE mnemonic for reports for scenes [18], the scenario of which was based on a previous real disaster event that forced the activation of the HDP; and

(3) knowledge of the protocols and actions described in the PEMAF disaster plan consisting of seven dichotomous questions (YES or NO); and, for each question in this session a brief explanation was requested, especially in case of an affirmative answer, in order to mitigate any potential falsehoods, biases and an observer-expectancy effect (the Rosenthal effect).

Interview questions and outline are reported as Additional file 1.

A panel of experts composed of three senior faculty members from the Research Center in Emergency and Disaster Medicine (CRIMEDIM), Università del Piemonte Orientale, Novara, Italy reviewed the structured interview instrument content for accuracy and provided appropriate modifications to ensure validity of the study.

### Ethical consideration

The participation in the study was voluntary, anonymous, and independent. Confidentiality of information was ensured and no financial incentive to participate in the study was offered. Verbal informed consent was obtained and the participants could withdraw from the poll at any time. Since all data were collected such that individual subjects could not be identified or exposed to risks or liabilities, the evaluation was deemed exempt from institutional review approval by the local Ethics Committee.

### Data analysis

The interview answers were entered in an online interview tool, hosted on SurveyMonkey (SurveyMonkey LLC, Palo Alto, California USA). Data were coded on a master sheet using a Microsoft Office Excel spreadsheet (Version 2003, Microsoft Corporation, Redmond, Washington USA). Frequencies were used to describe respondent characteristics.

The comparison of numerical variables was performed using the Mann–Whitney test for non- parametric data or the Kruskal-Wallis test as appropriate. Values of *p* < 0.05 were considered significant. Data are provided as Additional file 2.

## Results

**Fig. 1:**
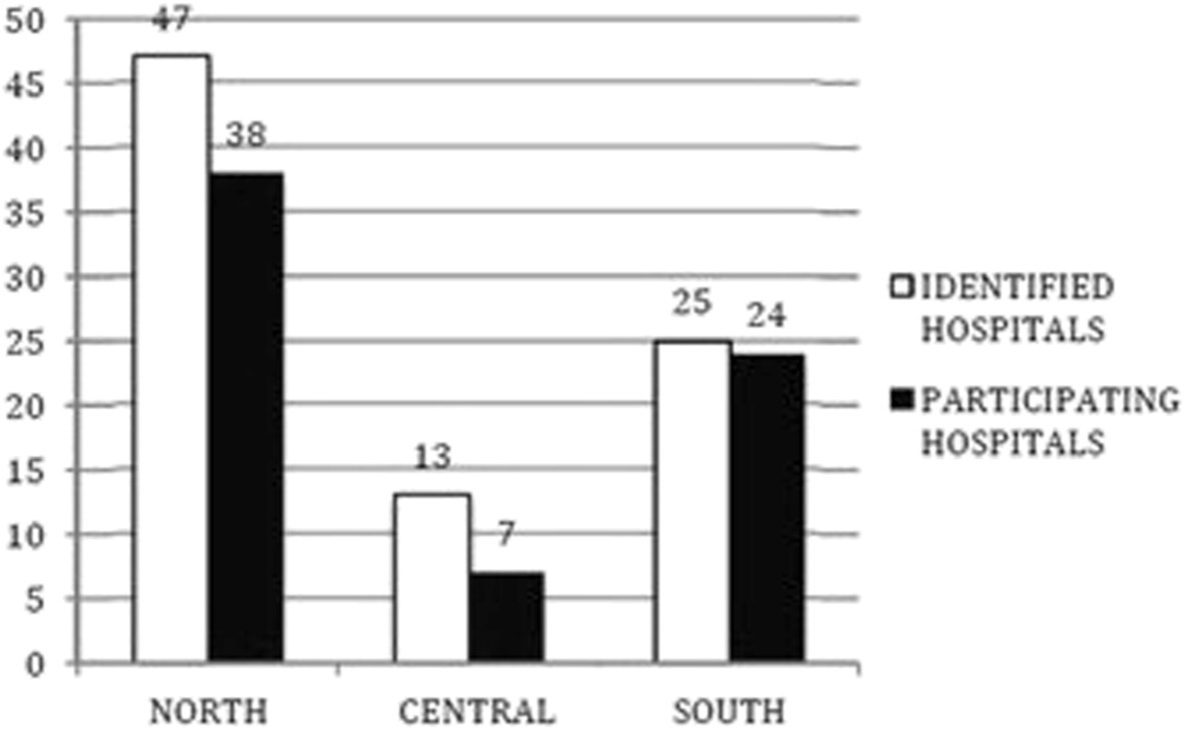
Geographical distribution of identified and participating hospitals by macro-areas of Italy

**Fig. 2:**
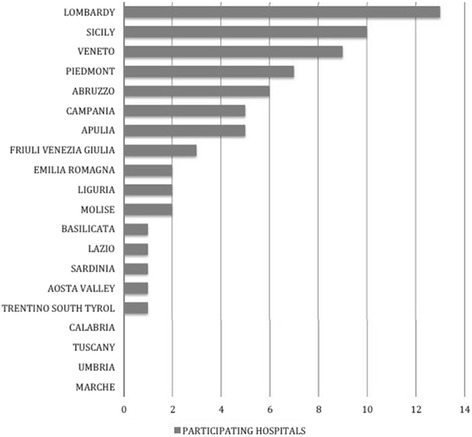
Geographical distribution of participating hospitals by Regions of Italy

Out of 352 EDs included in the Ministry of Health’s list, 85 (24 %) EDs were eligible for the interview sample. According to the Italian law D.Lgs. 70/2015, 52 of the included hospitals had second level EDs, and 17 first level EDs. The distributions of identified and participating hospitals through the country are represented in Figs. 1 and 2. Of the 69 (81 %) EDs that were reached, the physicians in charge agreed to undergo the telephone interview, with 30 at the first call attempt, 26 at the second and 13 at the third. Sixteen hospitals were excluded in the study: 6 for an explicit denial of call takers and 10 after the third call attempt resulted in no answer.

Most of the respondents were female (58 %), and the mean age was of 48 years. The most represented specialty was Internal Medicine (45 %). The characteristics of interviewed physicians are presented in Table 1. Only 45 % of participants reported to know what a HDP is and almost one third knew what an *action card* is which contains condensed information of vital disaster plan resources including critical contact numbers and personal information. In 41 % of the cases respondents believed to know who had the authority to activate the HDP and only 38 % knew who is in charge of all intra-hospital operations.

**Table 1:** Characteristics of participants

Demographics	
Mean Age ± SD (min-max)	48 ± 9 (32–61)
Female Gender, n (%)	40 (58 %)
Specialization, n (%)	
Internal Medicine	31 (45 %)
Emergency Surgery	8 (12 %)
Gastroenterology	7 (10 %)
Cardiology	4 (6 %)
Emergency Medicine	4 (6 %)
Geriatrics	4 (6 %)
General Surgery	3 (4 %)
Respiratory Medicine	3 (4 %)
Other Specialties	12 (17 %)
More than one specialty	7 (10 %)
Work experience in that ED (in years of service), n (%)	
0–1 year	5 (7 %)
2–4 years	5 (7 %)
More than 4 years	49 (86 %)
Prior attendance of disaster management courses, n (%)	
None	51 (74 %)
At least 1 course	18 (26 %)

Six different options were reported when asked who, according to the HDP, is appointed to activate the hospital plan. Both the Hospital Chief Medical Officer (HCMO) and the ED physician-on-duty were sighted in 10 out 28 cases. The command of all operations is taken by the ED Physician-on-duty in 10 hospitals, and by HCMO in another 7. The telephone was cited as the most used communication system to recall staff when the emergency status is declared (27 out of 55) and to find information about available beds (18 out of 50). Finally, nine different roles were identified to terminate the emergency status, HCMO being the most reported by responders (12 %).

The results are shown in details in Table *2*.

**Table 2:** Results of Section 2 and 3

Session 2: General knowledge about the HDP	YES	NO
1) what is the PEMAF	31 (45 %)	38 (55 %)
2) location of a copy of the PEMAF, for emergency reference and reading	23 (33 %)	46 (67 %)
3) what is an Action/Task Card	16 (35 %)	53 (65 %)
Session 3: Specific knowledge of the HDP	YES	NO
4) who activates the PEMAF	28 (41 %)	41 (59 %)
Hospital Chief Medical Officer	10 (35 %)	
Emergency Department physician-0n-duty	10 (35 %)	
EMS-OC	7 (25 %)	
Other	4 (13 %)	
Multiple answer	3 (9 %)	
5) who is in charge of intra-hospital operations	26 (38 %)	43 (62 %)
Emergency Department physician-on-duty	10 (38 %)	
Hospital Chief Medical Officer	7 (27 %)	
Crisis Unit	4 (15 %)	
Other	6 (20 %)	
Multiple answers	1 (3 %)	
6) Management of patients already admitted to the ED before PEMAF activation	27 (39 %)	42 (61 %)
Transfer/discharge by established protocol	13 (48 %)	
Less severe patient discharge	5 (19 %)	
Green codes assessed and discharged; yellow and red codes treated and held	3 (11 %)	
Other	6 (18 %)	
7) Way to recruit additional personnel	55 (80 %)	14 (20 %)
Telephone	27 (49 %)	
On call personnel list	14 (25 %)	
Switchboard	8 (15 %)	
Crisis Unit/ Head Nurse/ EMS-OC/ Hospital Chief Medical Officer to call personnel	4 (6 %)	
Respondent to personally call staff colleagues	2 (3 %)	
8) ED’s maximal patient management capacity in the first hour (by triage priority code)	11 (16 %)	58 (84 %)
Approximately more than 10	5 (45 %)	
Approximately less than 10	3 (27,5 %)	
It depends on the casualty severity	3 (27,5 %)	
9) Way to find information about bed number in the inpatient divisions/departments	50 (72 %)	19 (28 %)
By telephone	18 (36 %)	
Software	9 (18 %)	
Updated list (generic)	7 (14 %)	
“I personally call the departments”	5 (10 %)	
Other	9 (18 %)	
10) who terminates the emergency status	25 (36 %)	44 (64 %)
Hospital Chief Medical Officer	12 (48 %)	
EMS-OC	5 (20 %)	
Emergency Department physician on duty	3 (12 %)	
Other	10 (8 %)	
Multiple answers to the question	3 (12 %)	

Abbreviations: *PEMAF* Italian acronym for the Emergency Plan for Massive Influx of Casualties; *HDP* Hospital Disaster Plan; *EMS-OC* Emergency Medical Service Operation Center

Comparing the level of knowledge with the demographic descriptors, only prior attendance of disaster management courses evidenced a significant difference: respondents who had attended some instruction courses to enhance disaster management competencies were knowledgeable as compared to those with no previous training (*P* < 0,001). No other difference could be found between level of training, geographical distribution and emergency department level. Finally, the three General Surgeons scored higher than any other specialty because of the small numbers in each sample, and there were no significant differences in knowledge levels.

**Fig. 3:**
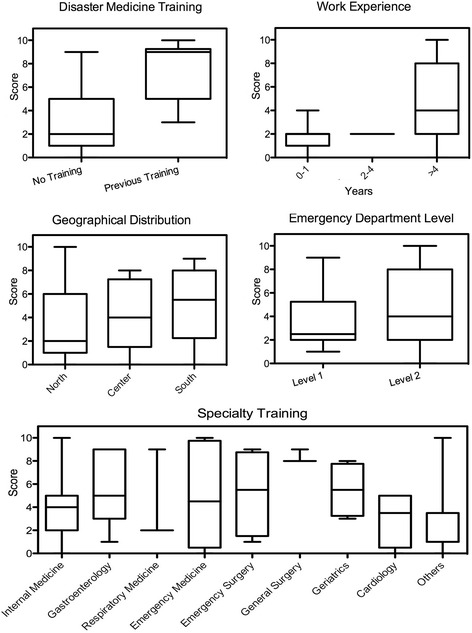
comparison of affirmative answer rates (score) to 10 questions on general and punctual aspects of PEMAF. **a** prior attendance of disaster management courses: affirmative answer rates of physicians who attended courses in disaster medicine was significantly higher (*p* < 0,001). **b**: work experience in that ED (years of service): no significant difference (*p* = 0,06). **c**: geographical distribution of respondents: no significant difference (*p* = 0,15); **d**: level of care provided by EDs: no significant difference (*p* = 0,70). **e**: specialty training of on duty staff: no significant difference (*p* = 0,24)

Figure 3 depicts the correct answers to the 10 questions (score) regarding different aspects of respondents’ demographics.

## Discussion

The current study evaluated the level of the knowledge-base of front line emergency physicians-on-duty regarding basic disaster planning and procedures. Adequate preparation of ED physicians has become particularly important following the problematic response seen during recent events [8, 19, 20]. It is therefore a key responsibility to verify the knowledge-base and subsequent behaviors of its frontline hospital employees since emergency services can hamper overall disaster management especially in the immediate response phase of crisis situations. Our results demonstrate the poor knowledge-base of basic hospital disaster planning concepts by EDs’ physicians-on-duty. About two thirds of those interviewed knew what a hospital disaster plan was or heard about it at least once, but only one third knew if there was a hard copy available for rapid consultation or w*hat an action* card was. The use of action/task cards, also known as standard operating procedures, has been recommended since 1972 and incorporate sufficient instructions, information and orders to guide, step by step, the members of the hospital staff [21]. Lennquist considers the task/action cards the most important component in hospital preparedness for major incidents [22]. It should be a major concern for hospital managers to find an adequate operational level method of distributing disaster policy and procedures to physicians and to make sure that physicians know and understand the important information.

Our study results are similar to previous reports that show inadequate disaster preparedness in Italian hospitals [23–25]. A pilot study made by D’Alessandro et al. involved a limited number of Italian hospitals in 2012 and demonstrated a state of low preparedness in complex emergency management. In particular, a written questionnaire was sent to hospitals, but the authors did not test directly the on-duty emergency consultants’ knowledge of HDP in real time through telephone interviews as was performed in this study. To the best of our knowledge, this study represents the first attempt to address the preparedness of Italian hospitals through determining the level of the actual knowledge-base of front line emergency department physicians-on-duty regarding basic disaster planning and procedures, during real working conditions.

This study also found that the on duty staff has poor knowledge of the function and roles in the required disaster response when the hospital emergency plan is activated. This is consistent with audit results of physician’s knowledge of major incident policies conducted by Carr and colleagues where less than 5 % of interviewed physicians were aware of their specific roles in such an event [26]. Some of the remaining questions revealed a lack of punctual preparation concerning specific procedures of the protocol, for example, the majority of EDs physicians did not know how additional personnel are summoned or how to find information about the number of available beds in the hospital wards. This may be due to the responders comparing the hospital disaster protocol with the more commonly understood daily working protocols and procedures. Of note, the study reveals an extreme overall variability of functions, roles and procedures in hospital disaster response. More than 5 different professionals or teams were reported to have the authority to initiate the HDP and its related actions and more than 10 had authority to terminate it. Seven different options were provided when the physicians were asked about who is in charge of all hospital disaster management. It is vital that the key function authority and responsibility be clearly specified, understood and standardized. Staff turnover today is an ongoing phenomenon, frequently on a daily basis, [27–29] and a variety of roles and procedures in exceptional situations such disasters could challenge staff response and affect overall outcome.

Finally, the study did not show any relevant difference in the level of knowledge-base among different specialties of on duty staff. The main reason of the lack of disaster medicine knowledge for health professionals might be that disaster medicine has rarely been included in Italian medical school curriculum and continuing medical education. In fact, only a small portion of the frontline emergency physicians enrolled in the study have attended courses in disaster medicine designed to enhance their competence and level of the required knowledge-base in hospital response. Of note, these responders evidenced significantly better knowledge-base suggesting a positive effect of disaster training on performance. Currently, there is an inadequate number of emergency medicine specialized physicians in Italy because this residency program has only been in existence since 2009.

The lack of education and training in disaster preparedness in the health systems at the EU level was recently denounced by Djalali et al. [6]. Promoting and enhancing the training capacity in the field of disaster medicine is one of the ‘call-to-action requirements’ requested by the international community. In forthcoming research, CRIMEDIM plans to better assess the deficiencies in major incident management and explore how to increase the level of hospital disaster preparedness at the country level.

### Limitations

This study had several limitations. Not all the EDs were reached and participated in the study due to the reasons previously stated. Therefore the results have to be interpreted carefully, and the conclusions cannot be extrapolated to every hospital or to every emergency physician in Italy.

However, this is the first study in Italy which attempts to address the hospital preparedness through determining the critical level of the disaster preparedness knowledge-base of frontline emergency physicians on duty at EDs regarding basic disaster planning and procedures. In addition, even though the structured interview and its content was internally validated based on consensus of the experts, it was never tested for reliability. As interviews in this study were completed by staff in between their daily ED duties, stress, exhaustion and time interviewed were factors in answering those questions, all of which understandably need more time to analyze and deeply think through before answering.

In conclusion, the study reveals that a low level knowledge-base of Italian frontline emergency physicians-on-duty at EDs with regard of expected HDP response to major incidents and disasters. The study also shows an high variability of functions, roles and procedures reported in local hospital disaster plans exposing the health staff to additional challenges. These findings should alert hospital managers, regional and national authorities to do more in disaster preparedness field as well as periodic training and unannounced testing. Building the knowledge-base of emergency department first responders and health staff is a necessary first step in disaster risk reduction and management. It is the hope that this study will prompt similar research within the global framework of emergency departments in other countries.

## Additional files

Additional file 1:Table S1. Description of Interview. (DOC 51 kb) Additional file 2: Availability of data and materials. (DOC 23 kb)

## Abbreviations

HDP: Hospital disaster plan
EDs: Emergency departments
PEMAF: Emergency Plan for Massive Influx of Casualties
HCMO: Hospital Chief Medical Officer
