# Supplementary material for: Assessment of disaster preparedness among emergency departments in Italian hospitals: a cautious warning for disaster risk reduction and management capacity
Source: Scand J Trauma Resusc Emerg Med. 2016 Aug 15;24:101. doi: 10.1186/s13049-016-0292-6 (PMC4986169; doi:10.1186/s13049-016-0292-6)
Supplement: Additional file 1:Table S1. — Description of Interview. (DOC 51 kb) [file 13049_2016_292_MOESM1_ESM.doc]

Additional File 1: Description of Interview

| ***Session 1: Demographics*** | | |
| --- | --- | --- |
|  | **1** | Age |
|  | **2** | Gender |
|  | **3** | Specialization |
|  | **4** | ED working experience in years |
|  | **5** | Attendance at disaster management courses |
| ***Session 2: General knowledge about the HDP*** | | |
|  | **6** | “Do you know what the PEMAF is?” |
|  | **7** | “Do you know where a copy of the PEMAF, for emergency reference and reading, is located?” |
|  | **8** | “Do You know what an Action/Task Card is?” |
| ***Simulated Call from EMS Operation Center*** | | |
| *a brief description of an incident was provided according to the METHANE mnemonic for reporting for scene data*  as following: | | |
| | **M** | Major incident | declared | | --- | --- | --- | | **E** | Exact location | 5 km | | **T** | Type of incident | Train-on-train collision | | **H** | Hazards | none | | **A** | Access | road | | **N** | Number of injured | approximately 300 people, 30 deceased | | **E** | Emergency services | ambulances, civil protection | | *Additional Information.*   - Estimated arrival time of first ambulance to the ED: 10 minutes. - Most prevalent injuries: burns and trauma casualties. | | | | | |
| ***Session 3: Knowledge about protocols & actions of the HDP*** | | |
|  | **9** | “Do you know who activates PEMAF?” |
|  | **10** | “Do you know who takes command of intra-hospital operations?” |
|  | **11** | “Do you know how to manage patients already admitted to the ED before PEMAF. activation?” |
|  | **12** | “Do you know how additional personnel are located?” |
|  | **13** | “Do you know what is the ED's maximal casualty management capacity in the first hour as listed by the triage priority code?” |
|  | **14** | “Do you know how to find additional information concerning the number of available beds in the various hospital divisions?” |
|  | **15** | “Do you know who declares the *all clear* status?” |

Description of interview. Abbreviations: PEMAF: Italian acronym for the Emergency Plan for Massive Influx of Casualties; HDP: Hospital Disaster Plan.
