# Supplementary material for: Assessment of disaster preparedness among emergency departments in Italian hospitals: a cautious warning for disaster risk reduction and management capacity
Source: Scand J Trauma Resusc Emerg Med. 2016 Aug 15;24:101. doi: 10.1186/s13049-016-0292-6 (PMC4986169; doi:10.1186/s13049-016-0292-6)
Supplement: Additional file 2: — Availability of data and materials. (DOC 23 kb) [file 13049_2016_292_MOESM2_ESM.doc]

The dataset supporting the conclusions of this article is available in the Dropbox repository:

[https://www.dropbox.com/s/35m4eti6dlinktr/PEMAF%20Raw%20Data.xls?dl=0](https://www.dropbox.com/s/35m4eti6dlinktr/PEMAF Raw Data.xls?dl=0)
